# Pro Free Will Priming Enhances “Risk-Taking” Behavior in the Iowa Gambling Task, but Not in the Balloon Analogue Risk Task: Two Independent Priming Studies

**DOI:** 10.1371/journal.pone.0152297

**Published:** 2016-03-28

**Authors:** Yann Schrag, Alessandro Tremea, Cyril Lagger, Noé Ohana, Christine Mohr

**Affiliations:** 1 Institute of Psychology, University of Lausanne, Lausanne, Switzerland; 2 Ecole Polytechnique Fédérale de Lausanne, Lausanne, Switzerland; Technion Israel Institute of Technology, ISRAEL

## Abstract

Studies indicated that people behave less responsibly after exposure to information containing deterministic statements as compared to free will statements or neutral statements. Thus, deterministic primes should lead to enhanced risk-taking behavior. We tested this prediction in two studies with healthy participants. In experiment 1, we tested 144 students (24 men) in the laboratory using the Iowa Gambling Task. In experiment 2, we tested 274 participants (104 men) online using the Balloon Analogue Risk Task. In the Iowa Gambling Task, the free will priming condition resulted in more risky decisions than both the deterministic and neutral priming conditions. We observed no priming effects on risk-taking behavior in the Balloon Analogue Risk Task. To explain these unpredicted findings, we consider the somatic marker hypothesis, a gain frequency approach as well as attention to gains and / or inattention to losses. In addition, we highlight the necessity to consider both pro free will and deterministic priming conditions in future studies. Importantly, our and previous results indicate that the effects of pro free will and deterministic priming do not oppose each other on a frequently assumed continuum.

## Introduction

Free will is a hotly debated research topic in psychology and other disciplines such as philosophy, the neurosciences, or physics. From a psychological viewpoint, free will can be understood as the capacity of an entity (humans in the current case) to make autonomous and conscious decisions [1]. A deterministic viewpoint, on the other hand, involves that any event can be fully explained by the physical state of the world preceding that event. Again, this viewpoint also applies to living entities such as humans. In the latter case, deterministic viewpoints assume that any human behavior can be fully explained by the person’s prior state (physical or mental). Thus, free will assumptions entail that we can choose between different ways of behaving in any given situation, while deterministic assumptions entail that we can only behave in one possible way, namely the one that is determined by the anteceding situation.

In experimental psychology, as in most scientific domains, a deterministic framework is almost taken as a given; the theories we develop aim for, and rely on, predictability and reproducibility. Consequently, the idea of free will seems contradictory to scientific theory leading to divergent viewpoints [2–7]. In the general population, however, the presumption of the existence of free will seems deeply engrained [8]. Members of the general population assume that they freely decide when taking daily decisions such as today’s meal, what they wear, or how they spend evening time. They also consider themselves free when taking more substantial decisions such as partner choice, professional goals, or how income is invested. This assumption of having free will is not necessarily a conscious process. Rather, when being in the decision making process, one may make a choice on the basis that the decision was freely taken rather than deterministically imposed. Yet, these beliefs in free will and resultant behavioral consequences do not seem fixed, but can fluctuate, as shown in numerous priming studies.

Vohs, Schooler and Baumeister, among others [8–14], showed that exposure to pro free will statements and deterministic statements can influence beliefs about free will and subsequent behavior. These priming paradigms entail that individuals are presented with different statements that are assumed to activate particular concepts or schemas. Here, participants are commonly presented with deterministic statements (e.g. “Science has proven that free will is an illusion”), pro free will statements (e.g. “I demonstrate my free will every day when I make decisions”), and / or neutral statements (e.g.”Oceans cover 71% of the earth’s surface”) before performing behavioral experiments. As compared to groups who had been primed with pro free will or neutral statements, participants who had been primed with deterministic statements cheated more [8], were less helpful and more aggressive [10], and showed less self-control [12]. In addition, deterministic statements had measurable effects on neuronal correlates of voluntary motor preparation, i.e. with reduced amplitude of the early readiness potential [11]. In sum, priming deterministic concepts seems to reduce the sense of conscientious behavior and agential control [9], and in turn hinder what appears to us as socially responsible and moral behavior [8]. Considering that behavior such as cheating [15] or lowered self-control [16] have been linked to enhanced risk-taking, we hypothesized that individuals primed with deterministic statements as compared to pro free will or neutral statements would demonstrate higher risk-taking.

In two subsequent studies, we tested this assumption. Firstly, we assessed behavior in the Iowa Gambling Task (IGT, [17]). Secondly, to test whether IGT findings would extend to other risk-taking measures, we assessed behavior in the Balloon Analogue Risk Task (BART, [18]) in a new population. The IGT was introduced by Bechara et al. [17,19] to test decision-making deficits in frontal lobe patients through risk-taking behavior. In this task, participants have to choose between four decks of cards. Unbeknownst to the participants, two desks are risky decks, which lead to negative overall outcomes in the long-term, and two are safe decks, which lead to positive overall outcomes in the long-term. Over several trials, in which participants receive feedback on their gains and losses, participants should learn to avoid the risky decks [19]. Using this task, it has been observed that patients suffering from frontal lobe lesions [17,20,21], pathological gamblers [22,23], and patients with drug addiction [24,25] failed to avoid the risky decks. A recent meta-analysis showed that healthy populations are not unequivocally good decision makers either, but that interindividual differences [26] as well as different heuristics [27] explain variation in the acquisition of risk avoidance strategies.

The BART is a well-established measure to assess risk-taking behavior [18,28]. Participants are presented with an empty balloon on the computer screen. Via button presses, they inflate the balloon. With each button press, the balloon may either grow or explode. The participant is asked to maximize the number of button presses for which the balloon is growing and not exploding. Participants receive a point for each button press for which the balloon is not exploding. In case the balloon explodes, the participant loses all points of this trial. Thus, the participant has to decide when s/he stops inflating the balloon. The BART has been shown to be a sensitive measure of risk-taking behavior in adolescents and adults [18,29]. Performance in the BART has also been associated with risky sexual behavior [30] and sensation-seeking traits [18]. Thus, the literature would indicate that both the IGT and the BART are sensitive measures to assess risk-taking behavior. It is, however, worth mentioning that other studies [29] showed that the performances in the two tasks do not necessarily correlate, indicating that they may assess different aspects of risk taking behavior.

In the first study, we predicted that individuals who were primed with deterministic statements would choose more frequently and over longer periods of time the risky decks in the IGT than individuals who were primed with pro free will statements or neutral statements. Contrary to these predictions, we observed enhanced risk-taking behavior in the pro free will condition. Accordingly, we tested in the next study whether this finding would replicate using the BART, i.e. whether performance would result in more button presses in individuals who were primed with pro free will statements than with deterministic statements.

## Study 1

### Material and methods

#### Participants

We tested 144 students from the University of Lausanne (24 men). They had a mean (±SD) age (always in years) of 20.96 (± 3.33, range 18 to 38). Participants were recruited via personal contact or took part for course credits. In line with previous studies, we aimed for at least 30 participants per condition [31]. All participants provided written informed consent prior to participation (see also the procedure section for further ethical details).

#### Iowa gambling task

The task was run on the free IGT version provided on PEBL [32]. This version has the same structure as the original version of the IGT [17]. The PEBL IGT version can be found in the experiment library of the program. We translated the task instruction and description into French, but left the remaining parts of the task untouched. In brief, participants saw four decks of cards (A, B, C, D) which were placed next to each other on a computer screen. They were informed that each deck would award them virtual money. Yet, awards would differ by different amounts of gains and losses. At this point, not yet known to participants, gains and losses were set such that decks A and B were risky decks (high direct reward, but long-term losses) and decks C and D were safe decks (low direct reward, but long-term gains). Equally unknown to participants, decks varied in terms of overall gain frequency. As shown by [33,34], decks B and D have the highest gain frequency (9 net gains /1 net loss), followed by deck C (6.25 net gains / 2.5 draws / 1.25 net losses) and finally deck A (5 net gains / 5 net losses). The complete gain / loss sequence of each deck can be found in Table I in [33]. At this point, participants were informed that they have 100 choices with the ultimate goal of being awarded the highest possible amount of virtual money. In order to maximize motivated behavior [35], we told participants that the three best performers would receive gift vouchers of CHF 40.-, CHF 30.-, and CHF 20.-, respectively. After having received this information, participants were instructed to choose for each trial one of the four decks (moving the cursor to the respective deck indicating the choice by a mouse click).

#### Belief induction procedure

We performed a Velten-like procedure [36] for the priming procedure. Firstly, we translated the priming statements originally used by Vohs and Schooler [8] from English into French (the English version was provided by Dr. Vohs). Subjects were randomly allocated to one of the three priming conditions, i.e. the deterministic, pro free will, or neutral condition. For each priming condition (see S1 File for the full list of priming statements in French and English), participants were exposed to 15 sentences of deterministic statements (e.g. “Science has demonstrated that free will is an illusion”), pro free will statements (e.g. “I demonstrate my free will every day when I make decisions”), or neutral statements (e.g. “Oceans cover 71% of the earth’s surface”). More concretely, participants were asked to read 15 sentences within a minute each. They were also told that they should remember these sentences for a later price draw (performances of all participants would be compared along with IGT performances). The price draw took place according to the IGT score. Thus, recall performance of the sentences was irrelevant (was not assessed). This procedure was chosen to enhance focus and integration of semantic content of the sentences.

#### Overall procedure

Swiss Law does not require a specific ethic confirmation for these types of study. Yet, studies are required to follow the guidelines of the Helsinki Declaration [37]. When participants arrived to the lab, they were presented with an information sheet concerning the procedure of the experiment and ethical information in line with the guidelines of Helsinki. In particular, participants were informed on their rights as participants, *e*.*g*. that they could withdraw at any time and that their data would be treated anonymously (only demographic information such as sex, age, and education would be recorded). After receiving this information, participants signed their informed consent in paper format including their willingness that we can use their anonymous data for scientific purpose. In individual testing rooms, participants first filled in the demographic self-report questionnaire. Subsequently, they followed the on-screen instructions which led them from the belief induction procedure to the IGT. After study completion, we debriefed participants first orally, noted their score for the voucher draw and provided them with a written debriefing sheet explaining in detail the study, the hypothesis, and expected outcome.

#### Data analysis

We removed one participant due to missing demographic information. We also removed participants who selected the same decks almost exclusively throughout the experiment. Accordingly, we removed 4 participants (1 man) who showed highly repetitive behavior, i.e. they clicked on the same type of deck (safe or risky) for at least 4 consecutive blocks (80 clicks) up to the full 5 blocks (100 clicks). We kept the remaining 139 participants (23 men) for final analysis.

For the actual analysis, we divided the 100 trials into 5 blocks of 20 consecutive trials (as done originally in [18]). For each of these blocks, we calculated the percentage of safe deck choices (across C & D). We subjected this percentage score to a 3x5 mixed design ANOVA with priming conditions (pro free will, deterministic, neutral) as between-subject measure and IGT blocks (1,2,3,4,5) as repeated measure. Gender differences have been reported for risk-taking behavior [38], but have been of no interest to free will priming studies (e.g. [8,12]). Therefore, to control for potential gender effects on IGT performance, we added gender as a fixed factor. An exploratory, full factorial ANOVA showed that the interaction between gender and conditions was neither significant, *F*(2,133) = .288, p > .05, η_p_^2^ = .004, nor did it change the direction of the results. Thus, the interaction term was not further considered in order to obtain the most parsimonious model while still controlling for an overall gender effect [39]. Given the potential interest in gain frequency analysis, we calculated the percentage of high gain frequency decks (across B & D, see also [33,34]) for each block. This percentage score was subjected to a second, analogue 3x5 ANOVA with priming conditions (pro free will, deterministic, neutral) as between-subject measure and IGT blocks (1,2,3,4,5) as repeated measure. Individual deck performance was also evaluated (see also [40]). Participants have a total of 100 choices (across decks). Thus, the selection of a particular deck impacts the possibility to select another deck. To account for this codependency between the four decks, we calculated two proportion indices: 1) proportion of deck A choices from the bad decks (A / (A + B)); 2) proportion of deck C choices from the good decks (C / (C + D)). For each proportion, we performed separate 3x5 ANOVAs with priming as between-subject measure and blocks as repeated measure.

We report effect sizes throughout (partial eta squared for ANOVAs, Cohen’s d for pairwise mean comparisons). Post-hoc comparisons on significant main effects were performed using LSD tests. All *p*-values were two-tailed, the *α*-level was set at .05.

### Results

#### Participants

A Chi-square comparison on the number of men and women in the three priming conditions showed that they were equally distributed between conditions with 38 women on 45 participants for deterministic condition, 39 on 49 for pro free will condition and 39 on 45 for neutral condition, chi-square (*df* 2) = .898, *p* > .50, *V* = .084. A one-way ANOVA on age with priming conditions as the between-subject measure was not significant, *F*(2, 136) = 2.779, *p* > .50, η_p_^2^ = .040.

#### Performance in the IGT

**Fig 1:**
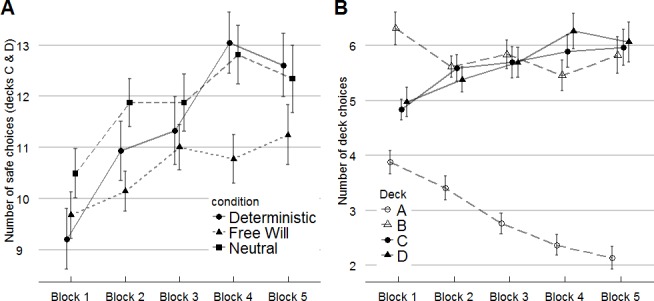
Performance in the IGT. A. Mean proportion of safe choices for five consecutive blocks of 20 choices each. Results are shown for priming conditions, separately. B. Mean proportion of deck choices (A, B, C, D) for five consecutive blocks of 20 choices each. The proportions are averaged over the three priming conditions. The figure shows that deck A was avoided (irrespective of priming condition, see result section). In both cases, vertical bars denote one standard error of the mean.

The ANOVA on the percentage of safe decks (C & D) showed a significant main effect of gender, *F*(1, 135) = 5.701, *p* = .018, η_p_^2^ = .041. Men chose safe decks less often (51.09±11.01, 95% *CI* [46.33, 55.85]) than women (57±10.01, 95% *CI* [55.16, 58.84]). The main effect of priming conditions was significant too, *F*(2, 135) = 3.343, *p* = .038, η_p_^2^ = .047. The main effect of priming condition did not change when leaving gender out of the model, *F*(2, 136) = 3.715, *p* = .027, η_p_^2^ = .052. Participants in the pro free will condition had a lower percentage of safe choice (52.84±8.84, 95% *CI* [50.3, 55.38]) than participants in both the deterministic (57.91±10.01, 95% *CI* [54.90, 60.92]) and neutral (57.6±11.61, 95% *CI* [54.11, 61.09]) priming conditions. The main effect of blocks was also significant, *F*(4, 540) = 8.014, *p* = .000, η_p_^2^ = .056. Post-hoc LSD pairwise comparisons showed that participants in the pro free will condition chose the safe decks significantly less frequently than those in both the deterministic priming condition, M*diff* = -4.809, 95% *CI* [-8.901, -.717], *p* = .022, *d* = .28, and the neutral priming condition, M*diff* = -4.376, 95% *CI* [-8.475, -.277], *p* = .037, *d* = .25. There was no significant difference between the deterministic priming condition and the neutral priming condition, M*diff* = .433, 95% *CI* [-3.741, 4.606], *p* > .05, *d* = .03 (Table 1). Finally, we observed no interactions between priming conditions and blocks, *F*(8, 540) = 1.303, *p* > .05, η_p_^2^ = .019 (Fig 1A), and between gender and blocks, *F*(4, 540) = 1.209, *p* > .05, η_p_^2^ = .009.

**Table 1:** Mean and standard deviation for each deck choices represented by conditions and by blocks.

Condition	Deck	Block 1	Block 2	Block 3	Block 4	Block 5	Total	Decks
**Deterministic**	A	3.89± 2.15	3.12± 2.22	2.4± 1.43	1.89± 1.23	1.89± 2.04	13.18± 5.13	**CD**
	B	6.34± 2.96	5.89± 2.12	6.32± 3.59	4.96± 2.92	5.43± 3.30	28.92± 8.21	57.91± 10.01
	C	4.83± 2.24	5.83± 2.08	5.60± 3.40	6.65± 3.75	6.06± 4.20	29.49± 10.01	**BD**
	D	4.96± 2.85	5.18± 2.26	5.69± 3.07	6.52± 3.73	6.09± 3.83	28.43± 9.68	28.67± 4.74
**Pro free will**	A	3.96± 2.00	3.56± 2.07	2.8± 1.71	2.64± 1.71	2.37± 1.96	15.31± 5.87	**CD**
	B	6.37± 3.64	6.31± 1.98	6.21± 2.43	6.60± 3.31	6.39± 3.87	31.86± 8.61	52.84± 8.84
	C	4.66± 1.77	5.25± 2.53	5.29± 2.08	5.43± 2.50	5.76± 3.52	26.37± 7.85	**BD**
	D	5.03± 2.82	4.9± 2.21	5.72± 2.77	5.35± 2.32	5.49± 3.23	26.47± 6.79	29.16± 4.57
**Neutral**	A	3.65± 1.71	3.36± 1.96	2.87± 1.80	2.27± 1.65	1.80± 1.61	13.94± 5.88	**CD**
	B	6.29± 3.03	5.14± 2.03	5.63± 2.80	5.25± 3.26	6.18± 4.14	28.47± 10.49	57.6± 11.61
	C	5.18± 2.03	5.56± 2.54	6.09± 3.50	5.74± 3.37	5.63± 2.88	28.18± 8.05	**BD**
	D	4.89± 2.99	5.96± 2.37	5.43± 2.69	6.76± 4.24	6.40± 4.70	29.43± 12.00	28.94± 3.69

The last column indicates the total percentage of choices by condition, grouped by safe decks (CD) and high gain frequency decks (BD)

The ANOVA on the percentage of high gain frequency decks (B & D) showed a significant main effect of blocks, *F*(4, 540) = 4.049, *p* = .003, η_p_² = .029, no main effect of gender, *F*(1, 135) = 2.596, *p* > .05, η_p_² = .019, no main effect of conditions, *F*(2, 135) = .116, p > .05, η_p_² = .002, and no interaction between blocks and conditions, *F*(8, 540) = 1.016, *p* > .05, η_p_² = .015 (Table 1).

The ANOVA on the A/(A+B) proportion index showed a significant main effect of blocks, *F*(4, 540) = 10.702, *p* < .000, η_p_^2^ *=* .073 (see Fig 1B), but no significant main effects of Gender, *F*(1, 135) = .289, *p* > .05, η_p_^2^ *=* .002, or condition, *F*(2, 135) = .138, *p* > .05, η_p_^2^ *=* .002. No significant interactions were found between blocks and gender, *F*(4, 540) = 1.817, *p* > .05, η_p_^2^ *=* .013, or blocks and condition, *F*(8, 540) = .539, *p* > .05, η_p_^2^ *=* .008.

The ANOVA on the C/(C+D) proportion index showed no significant main effect of blocks, *F*(4, 540) = 1.630, *p* > .05, η_p_^2^ *=* .012, no significant main effects of Gender, *F*(1, 135) = .077, *p* > .05, η_p_^2^ *=* .001, or condition, *F*(2, 135) = .338, *p* > .05, η_p_^2^ *=* .005. No significant interactions were found between blocks and gender, *F*(4, 540) = 1.290, *p* > .05, η_p_^2^ *=* .009, or blocks and condition, *F*(8, 540) = .533, *p* > .05, η_p_^2^ *=* .008.

## Study 2

### Material and methods

#### Participants

We tested 270 participants (101 men) with a mean age of 29.46 (± 14.1, range 18 to 79). Participants were recruited via personal contact, Internet, or took part for course credit. We recruited participants over a two weeks period after which the website was closed. To match our sample size to the sample size of study 1 and sample sizes commonly assessed with the BART (e.g. [18]), we aimed for at least 45 participants per priming condition (a number we easily had achieved). Before entering the experiment online, all participants provided informed consent prior to participation via button press after having received written study information (see also the procedure section for further ethical details). All data were recorded anonymously and participants were not traceable from the recorded information.

#### Self-report measures

To measure the effect of the priming procedure, we used different questionnaires to assess belief in free will and belief in determinism. To measure belief in free will, we used the Personal Will subscale of the Free Will and Determinism scale, e.g. “*I actively choose what to do from among the options I have*” (called Personal Will subscale from now on; [41]). We added the determinism beliefs subscale from the FAD-plus (called Determinism subscale from now on; [42]) to measure belief in determinism, e.g. “*People’s biological makeup determines their talents and personality”*, adding up to a total of 15 items. Participants replied to each item on a 5-point Likert scale ranging from 1 (entirely disagree) to 5 (entirely agree). Our French translation of these scales can be found in S2 File.

#### The BART

To allow online testing, we used a script written in JavaScript. Our BART version is slightly modified to the original BART [18]. Over the first slides, participants received the written information that their goal would be to reach a maximum score in a task in which they had to inflate a balloon through consecutive button presses. They could read that the balloon would inflate with each button press and also that the balloon could explode with each button press. It was specified that they would receive a score of 1 for each button press for which the balloon remained intact, and would lose all points of the current trial if the balloon exploded. They were informed that it was their choice when to stop inflating the balloon.

In the actual experiment, we presented a red small balloon in the middle of the computer screen. To the right, participant’s total score was presented. After each button press, the balloon got bigger or exploded. If the balloon exploded, no score was added to the total score. A new trial was initiated by presenting again the original balloon. If the participant decided to finish the trial, s/he could click on another button presented just below the balloon. The score of this trial was added to the total score. A new trial was initiated by presenting again the original balloon.

Overall, participants performed 30 trials. Specific to our online version, we asked participants at the beginning of the task to activate the sound of their computer (the computer would generate an exploding sound when the balloon exploded). The sequence of how many button presses would lead to the explosion of the balloon was pseudorandom, i.e., it was randomly generated, but remained constant between participants. Part of this pseudorandomization involved that the balloons of the first two trials would not instantly explode (this might lead to overtly prudent risk-taking behavior), but only after at least 10 button presses. The exact trial sequence, i.e., the maximum number of button presses before the balloon exploded, was 11. 15, 9, 8, 17, 11, 4, 14, 6, 13, 16, 2, 10, 9, 9, 7, 11, 11, 16, 13, 8, 17, 11, 8, 19, 18, 12, 13, 12 and 5. Overall, participants could make in average 11.17 button presses (range 2 to 19) before the balloon exploded.

We recorded the mean number of button presses for which the balloon did not explode (also called “adjusted number of pumps” as in the original study, see [18]) as our main measure of risk-taking behavior. Time between button presses and the number of exploded balloons were also recorded, but have not been considered superior as measures to the adjusted number of pumps [18].

#### Belief induction procedure

In the first study, we found that risk-taking behavior did not differ between the neutral priming condition and the deterministic priming condition. We enhanced statistical power by focusing on the two crucial priming conditions (as observed in study 1), i.e. deterministic priming condition and pro free will priming condition. To further adjust the procedure to the online measurement setting, we applied some additional modifications to the priming procedure. For each priming condition, we showed participants 10 statements that we have chosen from the pro free will statements or the deterministic statements (see S1 File for the selected statements) already used in study 1. Each statement was presented for 30 seconds. Subsequent to each presentation, a response field appeared without time limit. Into this field, participants wrote in their own words what the statement meant. We considered these modifications advantageous for three reasons. Firstly, through the reformulations, we can assume that participants processed the priming sentences more profoundly as compared to when only reading them. Secondly, through the reformulations, we could check if participants had read and understood the sentences. Thirdly, through the reduction of priming procedure trials (15 to 10 statements), we enhanced the likelihood that participants would actually finish the experiment (shortening the overall task duration).

#### Overall procedure

As mentioned in study 1, Swiss Law does not require a specific ethic confirmation for these types of study. Yet, studies are required to follow the guidelines of the Helsinki Declaration [37]. When participants activated the online link, they were guided through information screens on which we provided information on the study, the procedure and ethical information in line with the guidelines of Helsinki. In particular, participants were informed on their rights as participants, *e*.*g*. that they could withdraw at any time, and that their data would be treated anonymously (only demographic information such as sex, age, and education would be recorded). We also informed participants that we would consider their continuation (button press) as informed consent including their willingness that we can use their anonymous data for scientific purpose. When participants continued, they received instructions on the actual experiment.

**Fig 2:**
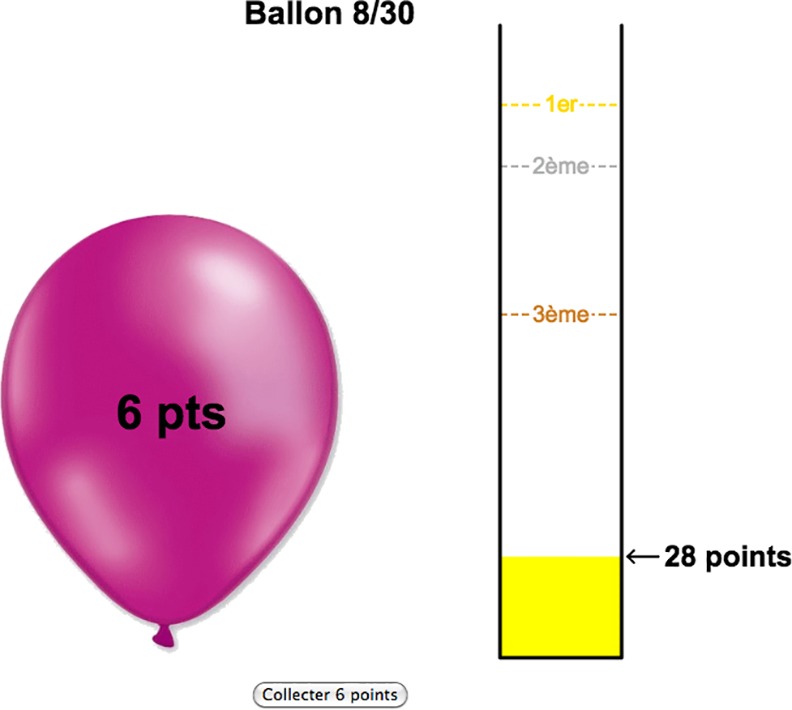
Screenshot during the online BART. On the balloon, the number of button presses (without the balloon exploding) during the ongoing trial was indicated. The size of the balloon increased with an increasing number of points (i.e. number of mouse clicks on the balloon). Below (“collecter 6 points”), the participant could see the number of points s/he would win if stopping the trial now (clicking on “collecter 6 points”). On the right, the participant could see the column rising; depending on how many points s/he collected across trials. To promote competiveness, participants saw the three best scores of “previous” players on the same column (1ier, 2ième and 3ième). Unknown to participants, these top scores had been set by us and were the same for each participant.

Once they had given their consent, subsequent pages would collect demographic information (age, gender, education, profession, study). At this stage, participants were randomly attributed to the pro free will or deterministic priming condition. After having reformulated the priming statements, participants performed the BART (see Fig 2). Finally, participants completed the short Personal Will subscale [41] and the Determinism subscale [42]. On the final pages, participants were asked whether they had activated the sound or not (the answer was not forced, unfortunately some participants did not answer) before being fully debriefed about the study.

#### Data analysis

From the original 270 participants, we excluded 14 (3 men) participants, because they gave poor, inadequate, or no reformulations of the priming statements. We kept the remaining 256 participants (98 men) for subsequent analysis.

To assess whether the priming statements had an effect on participants’ beliefs, we performed separate one-factor ANOVAs with priming conditions as the between-subject factor (pro free will, deterministic) on the i) Personal Will subscale scores and ii) Determinism subscale scores. Partial correlations, controlling for conditions, were also used to look at the possible link between the two beliefs subscales and risk-taking outside of any effect induced by the priming.

As mentioned in study 1, gender differences have been reported for risk-taking behavior [38], but have been of no interest to free will priming studies (e.g. [8,12]). Therefore, to control for potential gender effects on BART performance, we added gender as a fixed factor. An exploratory, full factorial ANOVA showed that the interaction between gender and conditions was neither significant, *F*(1, 252) = 1.940, *p* > .05, η_p_^2^ = .008, nor did it change the direction of the results. Thus, the interaction term was not further considered in order to obtain the most parsimonious model while still controlling for an overall gender effect [39].

Effect sizes are reported throughout. All *p*-values were two-tailed, the *α*-level was set at .05.

### Results and short discussion

#### Participants

There were an equal number of men and women in each priming condition with 75 women on 119 participants for deterministic condition and 83 on 137 for free will condition, chi-square (*df* 1) = .161, *p* > .05. A one-factor ANOVA on age with priming conditions as the between-subject measure was not significant, *F*(1, 254) = .460, *p* > .05, η_p_^2^ *=* .002.

#### Belief scale scores between priming conditions

An independent t-test showed that the Determinism subscale scores were higher in the deterministic priming condition (3.06± .571) than pro free will priming condition (2.79± .571), *t*(1, 254) = 3.861, *p* < .000, 95% *CI* [0.1353, 0.4170], *d* = .49. An analogue t-test on the Personal Will subscale score was not significant, *t*(1, 254) = -1.156, *p* > .05, 95% *CI* [-0.1673, 0.4352], *d* = .16.

#### Correlations between risk-taking and beliefs scales scores

Pearson correlations between belief questionnaire scores and the risk-taking score in the BART revealed no significant correlations between the Personal Will subscale score and the mean adjusted number of pumps (*r* = .066, *p* > .05), and between the Determinism subscale score and the mean adjusted number of pumps (*r* = .080, *p* > .05).

#### Risk-taking by conditions controlling for gender

The ANOVA with priming conditions and gender as fixed factor on the mean adjusted number of pumps revealed no main effect of priming conditions, *F*(1, 244) = 1.050, *p* > .05, η_p_^2^ = .004, but a significant main effect of gender, *F*(1, 253) = 37.125, *p* < .000, η_p_^2^ *=* .128. Men made more button presses (8.024±1.767, 95% *CI* [7.684, 8.356]) than women (6.696±1.636, 95% *CI* [6.433, 6.952]).

#### Power analysis and null results

In the current study, we did not replicate the observation that risk-taking (as assessed with the BART) was enhanced in the pro free will priming condition as compared to the deterministic priming condition. We do not think that this lack of significant difference results from the testing of an uncommon sample. In both studies, we replicated that men take more risks than women [18,38,43,44]. Moreover, we can infer on efficient priming effects based on the questionnaire data, because the deterministic priming condition group showed higher deterministic beliefs than the pro free will priming condition group.

A posteriori, to help with the interpretation of the non-significant result of study 2, we conducted a Bayesian ANOVA using JASP [45] and a power analysis using G*Power [46]. The Bayesian ANOVA was considered informative, because a non-significant result using classical inferential statistics does not suffice to endorse the null hypothesis [47]. This analysis supported the null hypothesis with a Bayes factor of *BF01* = 4.527 for the H0 and a Bayes factor of *BF10* = 1 / 4.527 = .221 for the H1. According to Jeffreys, this BF01 value suggests that the current data is about 5 times more probable under the H0 than H1, which can be considered as moderate evidence for the H0 (see [48] for further details on Bayes Factor interpretation). With regard to the power analysis, we took the effect size of our first study as an indicator (η_p_^2^ *=* .*047*, transformed into a Cohen’s *f* = .222), applied an alpha-level of .05, and our sample size of 256 participants. Using these values, we obtained a statistical power of 0.94278. This value represents an approximate 5.72% chance of having a type 2 error in our results, which is comfortably below the recommended maximum of 20% [49]. Truly, null results are the rarity rather than the norm in published studies. We nevertheless believe that they are important and of theoretical interest (see also [50,51]), especially when studies have sufficient statistical power minimizing type 2 errors (missing an existing effect). We suggest that our (null) results here are informative as such and also when discussing the results of both studies.

## General Discussion

We investigated whether individuals who had been exposed to deterministic statements as compared to free will or neutral statements would show higher risk-taking. This prediction was tested in two independent studies. In the first study, we investigated whether individuals would chose more risky decks (potential short-term gains, but overall long-term negative outcomes) over time in the IGT when having been primed with deterministic statements as compared to free will or neutral statements. Contrary to our initial predictions, participants showed higher risk-taking behavior in the pro free will condition as compared to both the deterministic and neutral priming condition. To test whether our results hold true for risk-taking more broadly, we performed a subsequent study using the BART. Here, however, no significant differences were found between pro free will and deterministic priming conditions. We do not assume that results reflect on aberrant behavior as such, because we replicated that participants increasingly chose safe decks in the IGT [19,52], men as compared to women showed higher risk-taking behavior in both tasks (see [38] for a meta-analysis on gender differences in risk-taking), and that Determinism subscale scores were higher in the deterministic than free will condition in the BART study. Our theoretical framework, originating from studies on free will (and deterministic) beliefs, does not offer an obvious explanation for these unexpected results [8,10,12,13]. It is not apparent that statements thought to bolster responsibility and feeling of freedom should promote risk-taking behavior. We, thus, discuss alternative explanations that could explain our findings focusing on theoretical frameworks originating in the decision making literature instead.

Historically, the IGT was introduced as a behavioral correlate supporting ideas conveyed in the Somatic Marker Hypothesis (SMH, [17,19,52]). The SMH implies that the decision making process (including the one occurring during IGT performance) relies importantly on emotional markers that are expressed somatically. Seminal studies showed that healthy individuals use somatic markers (as indicated by skin conductance responses) when learning to choose the safe decks in the IGT [17,21], whereas patients with frontal lobe impairments lacked both anticipatory skin conductance response and increasing avoidance of risky decks. Bechara et al. [19] argued that patients failed to learn to avoid negative outcomes, because they failed to access or use the somatic markers reflecting such emotional states. The SMH strongly suggest that the capacity to use somatic markers is key to efficient IGT performance, therefore, indicating that the IGT does not measure solely risk-taking behavior, but also affective decision making [17,19,53]. To our knowledge, such a link has not been reported for the BART which has been understood as a measure assessing risk-taking behavior [18,29]. Also, performance in the IGT has been shown to be uncorrelated to performance in the BART [29] indicating that both tasks are sensitive to different aspects of behavior.

According to the SMH framework, pro free will priming might have reduced affective decision making by hampering access to somatic marker information leading, by inference, to worse IGT performance. As such, our priming effects in the IGT might result from changes to decision-making style rather than to changes in risk-taking behavior per se. Indeed, most of the sentences constituting the pro free will priming procedure aim to reinforce the belief in agency, control, and responsibility. Exposure to such statements may enhance rational thinking and diminish affective decision making.

Although this explanation could be possible, an alternative literature provides a different approach to the decision making strategy in the IGT [33,40], and by inference a different explanation. Lin et al., [33,40] looked at how often the different decks were chosen. They observed a surprisingly stable preference for deck B, although this is a risky deck (one can gain 100 points, but lose 1250). Consequently, these authors and others (see also [54]) focused on gain frequencies instead of long-term gains usually assumed for the SMH: decks A and C are low gain frequency decks, while decks B and D are high gain frequency decks (providing short-term, salient reinforcement). A different clustering occurs for the SMH, i.e. decks A and B are risky decks (long-term losses) and decks C and D are safe decks (long-term gains). Here, the same decks (in particular deck B) might impact decision making behavior, once in combination with deck A to test for the SMH and once in combination with deck D to account for the gain frequency model [33,34].

In our gain frequency analysis, we replicated that overall choice behavior supported the gain frequency model, i.e. deck A was selected less frequently compared to the other three decks [33,54]. We observed, however, no interaction with priming conditions. Thus, our pro free will priming effect was only found when the risky decks A and B had been grouped together. Although this grouping originates in the original theoretical framework of the IGT (and by inference the SMH, [19]), we do not accept these results as proof for the SMH. The SMH explanation of IGT performance has been questioned repeatedly [33,40]. Apart from having unfavorable long-term outcomes, decks A and B have higher gain values and higher losses than decks C and D. In that regard, the preference for risky decks could also be due to high attention to gains values and / or inattention to losses values (as seen in [54]).

In sum, participants, whatever the priming condition, showed choice behavior in accordance with the gain frequency model (lowest likelihood to choose deck A). In addition, participants in the pro free will priming condition preferred short-term, high gain value decks (A & B) when compared to the other two priming conditions. Depending on the theoretical framework, these results could be explained by affective decision making (SMH, [19]) or high attention to gains values and / or inattention to losses values [54]. In order to dissociate these possibilities, studies can use the Columbia Card Task to separate affective from deliberative risk-taking behavior [55] and / or use the Soochow Gambling Task [33] balancing gain frequencies and long-term gains. If our results replicate, participants should show again the known preferences for high gain frequencies in the Soochow Gambling Task (A and B, [33]), whatever the priming condition. Furthermore, if our priming results are due to high attention to gains values, participants in the pro free will priming condition should prefer deck A over deck B in the SGT. Thus, at equal gain frequencies, they should show a preference for the deck that has the highest gain value, regardless of the high loss value.

Finally, we would like to comment on the observation that the pro free will priming condition and not the deterministic priming condition differed in its behavioral effects. In the first studies on free will related priming [8], the deterministic priming condition figured as the “experimental” manipulation condition (lowered free will beliefs). Effects due to free will primes were expected to be comparable to those due to neutral primes, because the belief in free will had been reported to be already prevailing and eminent [8]. Consequently, many studies only used the deterministic priming condition and as a control condition the neutral priming condition ([8] (first experiment),[11–13]). In line with above reasoning, when a pro free will priming condition was added, the dependent behavioral variables did not differ between the pro free will and neutral priming condition ([8] (second experiment),[10]). In our first study, however, priming effects occurred for the pro free will priming condition as compared to the neutral and deterministic priming conditions. Thus, our behavioral differences only occurred because we had used a pro free will priming condition. The theoretical implications are that behavioral effects do not only occur when pro free will beliefs are challenged (using the deterministic priming condition), but also when they are reinforced by a pro free will priming manipulation.

Few independent studies also used a pro free will priming condition in addition to deterministic and neutral priming conditions ([8] (second experiment),[10]). These authors found that behavior was comparable for the pro free will and neutral priming conditions, with behavior differing for the deterministic priming condition. The comparable behavior contrasted with higher self-reported belief in free will in the pro free will condition and lower self-reported beliefs in the deterministic condition compared to the neutral condition [8]. In our study, on the other hand, comparable behavior was observed for the deterministic and neutral priming conditions, with behavior differing for the pro free will priming condition. These differences between studies might indicate that the effects of the pro free will and the deterministic priming conditions do not lie at opposite ends of a continuum, but result from different cognitive processes affecting different types of behaviors. Indeed, if the pro free will and deterministic priming conditions would truly have opposite effects, we should have seen such opposing behavior in studies using all three priming conditions. Future studies are needed to confirm or reject this hypothesis. We consider these observations important for future studies because they invite researchers to 1) reconsider the notion that behavior would not be affected by pro free will priming in general, and 2) always use both deterministic and pro free will priming conditions.

### Study challenges and limitations

Our results do not allow us to settle which cognitive mechanisms were at play in the pro free will priming condition as compared to the other priming conditions. We considered the affective decision making explanation [19,56] and the idea that pro free will priming influenced attention to gains and / or inattention to losses (as in [54]). Future studies are needed that account for these (and potentially additional) conjectures. Studies could empirically account for somatic markers (e.g. assessing skin conductance responses [19]), affective decision making (e.g. questionnaires and behavioral assessments [55]), and alternative risk-taking tasks that account for gain frequencies (e.g. [33,34,54]) as well as attention to gains and / or inattention to losses. Moreover, when systematically varying instructions (e.g. [57]), we could investigate why results in the IGT and BART differ, such as knowing about the consequences of one’s behavior a priori (BART) or having to learn it through experience (IGT). A careful look at priming effects per se would help determining which priming procedures are most efficient and should be favored in the future. This suggestion includes the possibility to apply the reformulation manipulation we applied to the BART study (see also [58]). Participants had to process each priming statement profoundly in order to reformulate its meaning using own words. When looking at these reformulations, we realized that 14 participants had not understood the sentences (we consequently excluded these participants).

Beyond the risk-taking measures as such, future studies should test participants of comparable demographic profiles, i.e. an equal number of men and women of comparable ages. In the IGT study, we tested psychology students in the laboratory resulting in many more female than male participants. In the BART study, we tested individuals online. We obtained data from the larger public resulting in a more balanced gender proportion. In addition, we tested participants in the laboratory in study 1 and online in study 2. While one could think that participants in study 2 did not properly perform the task (reducing the likelihood of priming effects), we do not think that this was the case (see reformulation manipulation in previous paragraph). Also, online experiments have repeatedly been shown to yield similar results to those in laboratory settings [59]. In our study too, we observed previously reported gender differences [38] in the laboratory (IGT) and online (BART).

In the BART study, we observed higher scores in the determinism subscale in the deterministic priming condition as compared to the pro free will priming condition. Thus, we felt it legitimate concluding that the priming manipulation had worked. On the other hand, scores of the free will subscale did not differ between priming conditions. We have not used these subscales in the IGT study. Thus, we do not know whether the priming manipulation in the IGT study should have also influenced free will beliefs and deterministic beliefs. According to some authors [8,10], one has to assume that free will beliefs should have been higher in the pro free will priming group (as compared to the other priming groups) in the IGT study. In such a case, priming effects on behavior would accompany analogue priming effects on self-report free will belief scores. If we would have had self-report questionnaire measures from both studies, we could have investigated whether pro free will priming effects were reflected in both IGT performance and free will belief scores. In that case, absent priming effects on BART performance might be due to insufficient pro free will priming effects. A final limitation is that participants could win monetary reward according to their IGT total score (the 3 best performers received a voucher). We provided no reward in study 2. Based on previous studies, we do not think that these differences in reward conditions could explain study results [35].

## Conclusion

We tested in two studies whether deterministic priming (as compared to free will or neutral priming) would enhance participants’ risk-taking behaviors. We found instead that participants were taking more risk in the IGT after a pro free will priming procedure. We observed no such differences in the BART. Having considered two different approaches to explain these results (long-term gains [19]; gain frequency [33]), we suggest that our priming effect can be explained by either hindered affective decision making (based on the SMH [19]) or altered attention to gains and / or to losses [54]. Important for future studies, researchers should firstly consider alternative risk-taking tasks balancing for long-term gains and gain frequency [33]. Moreover, they should neither treat a neutral priming condition and a pro free will priming condition as entirely analogue, nor a pro free will priming condition and a deterministic priming condition as necessarily opposites. Rather, they should consider that pro free will and deterministic priming may affect cognitive processes differently. Additionally, our results generally show that despite overall high levels of free will beliefs in the general population, a pro will free priming manipulation might affect behavior that is assessed in the IGT (whether affective decision making or attention to gains and / or inattention to losses).

## Supporting Information

S1 File Material for the priming procedure. (DOCX)

S2 File French translated questionnaires. (DOCX)
