# Supplementary material for: Pro Free Will Priming Enhances “Risk-Taking” Behavior in the Iowa Gambling Task, but Not in the Balloon Analogue Risk Task: Two Independent Priming Studies
Source: PLoS One. 2016 Mar 28;11(3):e0152297. doi: 10.1371/journal.pone.0152297 (PMC4809538; doi:10.1371/journal.pone.0152297)
Supplement: S1 File — (DOCX) [file pone.0152297.s001.docx]

**S1. Material for the priming procedure.** The original English version was created and provided by Dr. Kathleen D. Vohs. The French version was translated by us. Neutral, deterministic and free will statements used in study 1. Statements used in study 2 are marked with an asterisk.

**Original English version**

### Neutral Statements

1. Oceans cover 71% of the earth's surface.

2. Alkaline power cells generally work longer than ordinary batteries.

3. Monarch butterflies fly slowly but have been sighted hundreds of miles at sea.

4. The Olympics are held every four years.

5. Half a day's boat ride away from Athens lies the isle of Mykonos.

6. Sugar cane and sugar beets are grown in 112 countries.

7. Many of the mountain peaks in the Rockies are over 14,000 feet high.

8. The Appalachian Highlands are worn down mountains and plateaus stretching from the northern Alabama to the St. Lawrence River in Canada.

9. The greatest distance the earth is from the sun is 94,452,000 miles.

10. The Nile River in Africa is the world's longest river.

11. The Los Angeles metropolitan area is known for its complex system of highways.

12. Most appliances are guaranteed for a full year against defects.

13. Pocket calculators became common items only after 1970.

14. Inventories are most frequently taken either at the beginning or at the end of the month.

15. Organically grown foods are more popular in recent decades.

**Deterministic Statements**

1. *Ultimately, we are biological computers - designed by evolution, built through genetics, and programmed by the environment.
2. *The brain is a complex machine capable of carrying out extremely sophisticated behaviors.
3. Science has demonstrated that free will is an illusion.
4. *It is likely that scientists will eventually understand how the feeling of personal experience results from neurons firing in the brain.
5. *Everything a person does is a direct consequence of their environment and genetic makeup.
6. *Once scientists understand enough about the physical principles underlying behavior, they should be able to precisely predict a person’s future actions based solely on that person’s genetics and prior experiences.
7. *Our actions are determined by what we have experienced in the past combined with the specific genetic predispositions that we have.
8. *Like everything else in the universe, all human actions follow from prior events and ultimately can be understood in terms of the movement of molecules.
9. *A belief in free will contradicts the known fact that the universe is governed by lawful principles of science.
10. Our mental activities are exclusively the product of physical processes.
11. *Every action that a person takes is caused by a specific pattern of neural firings in the brain.
12. All behavior is determined by brain activity, which in turn is determined by a combination of environmental and genetic factors.
13. *People often claim that they have free will, but all they really have is the experience of making choices.
14. Just as science has shown that physical movement is merely forces of gravity combined with muscular force, scientists are now realizing that personal thoughts, feelings, and beliefs are similarly controlled by basic physical processes.
15. Even if some behaviors are not actually pre-determined, this does not mean there is free will, as random actions are no more under our control than are those caused by prior events.

**Free Will Statements**

1. *I demonstrate my free will every day when I make decisions.
2. *I am able to override the genetic and environmental factors that sometimes influence my behavior.
3. *I have feelings of regret when I make bad decisions because I know that ultimately I am responsible for my actions.
4. *I take personal pride in good decisions I have made in the past because I know that, at the time, I had the freedom to and could have made a bad decision.
5. Avoiding temptation requires that I exert my free will.
6. *Ultimately people cannot blame their own actions on anything other than themselves.
7. *I have free will to control my actions and, ultimately, to control my destiny in life.
8. *I am more than a robot that has been programmed by genetics and the environment, no matter what a few scientists claim.
9. *People are responsible for their behaviors because they have free will to control their actions.
10. *Our actions and thoughts are not simply the result of prior experiences.
11. By exerting their free will, people can and do overcome the negative effects of a dysfunctional environment.
12. It has been shown that mental experience cannot be completely reduced to physical causes.
13. *There are many things that science still cannot explain, so it does not trouble me that science cannot offer an explanation for free will.
14. Given that I have had personal experiences that science cannot explain, I also know that I have free will even if science cannot explain it.
15. By exerting my will, I overcome the physical factors that influence my behavior and experience true freedom.

**French translation**

### Neutral Statements

1. 71% de la surface de la terre est couverte par les océans.

2. Les piles alcalines fonctionnent généralement plus longtemps que les batteries ordinaires.

3. Les papillons monarques volent lentement, mais certains ont pourtant été repérés en mer à des centaines de kilomètres de la côte.

4. Les jeux olympiques ont lieu tous les quatre ans.

5. L’île de Mykonos se situe à un demi-jour de bateau d’Athènes.

6. La canne à sucre et la betterave sucrière sont cultivées dans 112 pays.

7. Beaucoup de sommets de montagnes dans les Rocheuses au Colorado dépassent les 4000 mètres.

8. Albert Einstein a été tour à tour de nationalité allemande, apatride, puis suisse et enfin il acquit une double nationalité suisse et américaine.

9. La distance la plus grande à laquelle la terre peut être du soleil est de 152’150’600 kilomètres.

10. Le Nile, en Afrique, est la rivière la plus longue du monde.

11. La région métropolitaine de Los Angeles est connue pour la complexité de son système d’autoroutes

12. Le physicien Stephen Hawking a reçu la médaille Franklin en 1981, de plus l’astéroïde Hawking a été nommé en son honneur.

13. Les calculatrices de poche sont devenues des objets communs seulement après 1970.

14. L’inventaire est en général effectué soit au début, soit à la fin du mois.

15. La nourriture « bio » est devenue de plus en plus populaire durant les dernières décennies.

**Deterministic Statements**

1. *En définitives, nous sommes des ordinateurs biologiques – conçus par l’évolution, construits génétiquement et programmés par l’environnement.
2. *Le cerveau est une machine complexe capable de produire des comportements extrêmement sophistiqués.
3. La science a démontré que le libre-arbitre était une illusion.
4. *Il est probable que les scientifiques finissent par comprendre que le sentiment lié à l’expérience subjective résulte en fait de l’activation des neurones dans le cerveau.
5. *Tout ce qu’une personne fait est une conséquence directe de son environnement et de son bagage génétique.
6. *Quand les scientifiques auront mieux compris les principes physiques sous-jacents du comportement, ils devraient pouvoir prédire précisément les actions futures d’une personne en se basant seulement sur les gènes de cette personne et ses expériences passées.
7. *Toutes nos actions sont déterminées par ce que nous avons vécu dans le passé combiné avec les prédispositions génétiques spécifiques que nous avons.
8. *Comme tout le reste dans l’univers, toutes les actions humaines sont la conséquence d’événements précédents et peuvent être comprises en termes de mouvement de molécules.
9. *Croire que le libre-arbitre existe contredit le fait établi que l’univers est gouverné par les principes de la science.
10. Nos activités mentales sont exclusivement le produit de processus physiques.
11. *Chaque action entreprise par une personne est causée par un agencement particulier de neurones dans le cerveau qui correspond à cette action.
12. Tous les comportements sont déterminés par l’activité du cerveau, qui est lui-même déterminé par l’interaction entre l’environnement et nos gènes.
13. *Les gens prétendent souvent être libres dans leurs choix, mais tout ce qu’ils ont réellement n’est que l’impression de faire des choix.
14. De la même façon que la science a démontré que le mouvement physique n’est que la combinaison des forces de la gravité et des forces musculaires, les scientifiques réalisent maintenant que la pensée, les sentiments et les croyances sont également contrôlés par des processus physiques basiques.
15. Quand bien même certains comportements ne sont en fait pas prédéterminés, cela ne veut pas dire que le libre-arbitre existe réellement, car les actions aléatoires ne sont pas plus sous notre contrôle que les actions causées par des événements externes à notre volonté.

**Free Will Statements**

1. *Chaque fois que je prends une décision, je prouve l’existence de ma liberté de penser.
2. *Si besoin est, je suis capable de passer outre les influences de l’environnement et de mes gènes pour prendre ma propre décision.
3. *Lorsque je prends une mauvaise décision, j’ai des sentiments de regrets, car je sais que je suis responsable de mes actions.
4. *Je tire une fierté personnelle des bonnes décisions que j’ai prises par le passé, parce que je sais qu’à ce moment-là, j’étais libre de mon choix et aurais pu prendre une mauvaise décision.
5. Pour éviter les tentations, je dois faire usage de mon libre-arbitre.
6. *En définitive, les gens ne peuvent s’en prendre qu’à eux-mêmes quant à la responsabilité de leurs actions.
7. *J’ai le libre-arbitre nécessaire pour être maître de mes actions et au final c’est moi qui contrôle ma destinée dans la vie.
8. *Quoiqu’en disent certains scientifiques, je suis plus qu’un robot programmé par la génétique et l’environnement.
9. *Les gens sont responsables de leurs comportements parce qu’ils ont le libre-arbitre nécessaire pour contrôler leurs actions.
10. *Nos actions et nos pensées ne sont pas uniquement le résultat de nos expériences passées.
11. Par la force de leur volonté les gens sont capables de surpasser les effets négatifs d’un environnement dysfonctionnel.
12. Il a été démontré que notre expérience mentale, notre volonté et notre comportement ne peuvent pas être réduis à des causes physiques.
13. *Il y a beaucoup de choses que la science ne peut toujours pas expliquer, donc cela ne me dérange pas que la science ne puisse pas fournir d’explication concernant le libre-arbitre.
14. En partant du fait que j’ai vécu des expériences que la science ne peut pas expliquer, je sais également que je suis libre dans mes choix quand bien même la science ne pourrait l’expliquer.
15. Par l’exercice de ma volonté, je peux dépasser les facteurs physiques qui influencent mon comportement pour atteindre une réelle liberté.
