# Supplementary material for: Pro Free Will Priming Enhances “Risk-Taking” Behavior in the Iowa Gambling Task, but Not in the Balloon Analogue Risk Task: Two Independent Priming Studies
Source: PLoS One. 2016 Mar 28;11(3):e0152297. doi: 10.1371/journal.pone.0152297 (PMC4809538; doi:10.1371/journal.pone.0152297)
Supplement: S2 File — (DOCX) [file pone.0152297.s002.docx]

**S2. French translated questionnaires.**

**FWD personal will subscale**

1. Je suis le/la responsable de mes décisions.
2. Je suis actif dans mes décisions parmi les options que j’ai.
3. Je suis le/la responsable de mes actions même lorsque les circonstances de ma vie sont difficiles.
4. Mes décisions sont influencées par un pouvoir supérieur.
5. Je conserve toujours mon libre-arbitre même quand mes choix sont limités par les circonstances extérieures.
6. Je décide comment agir dans telle ou telle situation.
7. Mes choix sont limités parce qu’ils s’inscrivent dans un plan qui me dépasse.
8. J’ai un libre-arbitre.

**FAD-plus determinism subscale**

1. Le caractère des enfants est déterminé par celui de leurs parents.
2. Le bagage biologique d'une personne détermine ses talents et sa personnalité.
3. L'environnement durant l'enfance détermine le succès dans la vie adulte.
4. Les psychologues et psychiatres vont finir par pouvoir expliquer tous les comportements humains.
5. Vos gènes déterminent votre futur.
6. Comme pour les autres animaux le comportement humain suit toujours les lois de la nature.
7. La science a montré que votre environnement passé créait votre intelligence actuelle et votre personnalité.
